# BCG Vaccination Induces *M. avium* and *M. abscessus* Cross-Protective Immunity

**DOI:** 10.3389/fimmu.2019.00234

**Published:** 2019-02-19

**Authors:** Getahun Abate, Fahreta Hamzabegovic, Christopher S. Eickhoff, Daniel F. Hoft

**Affiliations:** ^1^Division of Infectious Diseases, Allergy and Immunology, Department of Internal Medicine, Saint Louis University, St. Louis, MO, United States; ^2^Department of Molecular Microbiology and Immunology, Saint Louis University, St. Louis, MO, United States

**Keywords:** nontuberculous, mycobacteria, BCG, *avium*, abscessus

## Abstract

Pulmonary non-tuberculous mycobacterial (NTM) infections particularly caused by *Mycobacterium avium* complex (MAC) and *Mycobacterium abscessus* (MAB) are becoming major health problems in the U.S. New therapies or vaccines which will help prevent the disease, shorten treatment duration and/or increase treatment success rates are urgently needed. This study was conducted with the objective of testing the hypothesis that Bacillus Calmette Guerin (BCG), a vaccine used for prevention of serious forms of tuberculosis (TB) in children and adolescents in tuberculosis hyperendemic countries, induces cross-protective T cell immunity against *Mycobacterium avium* (MAV) and MAB. Human TB and NTM cross-protective T cells were quantified using flow cytometric assays. The ability of BCG expanded T cells to inhibit the intracellular growth of MAV and MAB was assessed in co-cultures with infected autologous macrophages. In both BCG-vaccinated and *M. tuberculosis* (Mtb)-infected mice, NTM cross-reactive immunity was measured using IFN-γ ELISPOT assays. Our results demonstrate the following key findings: (i) peripheral blood mononuclear cells from TB skin test-positive individuals contain MAV and MAB cross-reactive T cells, (ii) both BCG vaccination and Mtb infection of mice induce MAV and MAB cross-reactive splenic cells, (iii) BCG-expanded T cells inhibit intracellular MAV and MAB, (iv) CD4, CD8, and γδ T cells play important roles in inhibition of intracellular MAV and MAB and (v) BCG vaccination of healthy volunteers induces TB and NTM cross-reactive T cells. In conclusion, BCG-vaccination induces NTM cross-reactive immunity, and has the potential for use as a vaccine or immunotherapy to prevent and/or treat pulmonary NTM disease.

## Introduction

In North America, the incidence of pulmonary nontuberculous mycobacteria (NTM) is higher than the incidence of tuberculosis (TB) (1). In addition, the prevalence of multiple NTM infections and the mortality rates associated with NTM infections are increasing (2–5). A study of Medicare part B beneficiaries showed that the prevalence of NTM increased from 20 to 47 per 100,000 persons between 1997 and 2007, an increase of 8.2% per year (2). A more recent report estimated that the number of pulmonary NTM cases in the US increased by at least another two-fold between 2010 and 2014 (5). The causes for these increases in prevalence of pulmonary NTM are not known.

NTM can affect any organ in the body although pulmonary disease is most common in HIV-negative patients (6–8). Risk factors such as cystic fibrosis (CF), chronic obstructive lung disease (COPD), bronchiectasis and thoracic skeletal abnormalities make NTM, particularly *Mycobacterium avium* complex (MAC) and *M. abscessus* (MAB), deadly pathogens (9–12). MAC and MAB are the most common causes of pulmonary NTM (3, 6, 13, 14). Pulmonary MAC and MAB are difficult to treat for two major reasons. First, the treatment regimens are very long, requiring the use of multiple drugs for at least 18 months (15). Second, the failure and relapse rates may exceed 40% (16, 17). Therefore, strategies to improve the prevention and treatment of pulmonary NTM in high risk patients are needed.

Similar to *M. tuberculosis* (Mtb), MAC and MAB are primarily intracellular pathogens and cell mediated immunity plays a major role in protection (18, 19). Therefore, vaccine strategies for NTM should be similar to strategies employed for TB, relying mainly on inducing or boosting protective cell mediated immunity. Notably, there appears to be an overlap between protective immunity for TB and that of NTM. For instance, epidemiological studies indicate that BCG vaccination is associated with marked decreases in *Mycobacterium avium* (MAV) disease prevalence (20). Similarly, latent TB infection decreases the risk of NTM disease (21) further suggesting the importance of cross-protective immunity. However, the basis for this cross-protective immunity and cell types involved in cross protection are not known.

This study was conducted to identify NTM cross-reactive immunity induced by BCG vaccination in immunocompetent mice and humans, and to evaluate the protective capacity of cross-reactive T cells by measuring their ability to kill intracellular NTM.

## Materials and Methods

### Samples

Peripheral blood mononuclear cells (PBMC) were obtained by Ficoll-Paque (GE Healthcare, Piscataway, NJ) centrifugation of blood samples obtained from healthy purified protein derivative (PPD)-positive volunteers (*n* = 10). Only frozen PBMC were used. All PPD-positive volunteers had a history of either latent TB infection and/or BCG vaccination. The protocol for blood draw and use of samples was approved by the Saint Louis University Institutional Review Board (IRB), and informed consent was obtained from each volunteer.

In addition, PBMC harvested pre- and 43-days post-BCG vaccination from five volunteers who were enrolled in a previously published clinical study were used (22). All volunteers were healthy, 18–45 years old, BCG naive, HIV and hepatitis C negative, and had no latent TB infection based on negative QuantiFERON TB-Gold (Cellestis) results. All five volunteers received a single intradermal vaccination with TICE® BCG (Organon Teknika, Durham, NC) containing ~2 × 10^6^ colony forming units (CFU) in 0.1 ml saline over the deltoid muscle. Intradermal, not percutaneous, was used because of previous findings showing a better immunogenicity from intradermal vaccination (23). TB skin test was not performed after vaccination. However, all five volunteers had detectable BCG shedding between 4 and 85 days post-vaccination, with four volunteers having grossly ulcerative lesion at the vaccination site (22). Screening, BCG vaccination, blood draws and use of PBMC in the different assays followed the protocol approved by the Saint Louis University Institutional Review Board, Saint Louis. Research was carried out according to the principles of the Declaration of Helsinki. All volunteers signed written consent forms including permission for future use of their stored samples.

### Reagents

Connaught BCG, MAV (ATCC 700898), MAV-whole lysate (WL), MAB (NR-44261, BEI Resources) and MAB-WL were used for *in vitro* expansion of mycobacterium-specific T cells. The following antibodies from BD Bioscience were used for flow cytometric analyses: anti-γδ T cell receptor (TCR) antibody-phycoerythrin (PE) (clone 11F2), anti-γδ TCR APC (Clone B1), anti-αβ TCR antibody-fluorescein isothiocyanate (FITC) (clone B3), anti-CD3 antibody-peridinin chlorophyll protein (PerCP) (clone SK7), anti-CD4 Pacific Blue (clone RPA-T4), anti-CD8 antibody–PE-Cy7 (clone RPA-T8), anti-IFN-γ APC antibody-Alexa Fluor 700 (clone B27), and anti-granzyme (GZM)-A antibody-FITC (clone CB9). Carboxyfluorescein succinimidyl ester (CFSE) was obtained from Molecular Probes (Eugene, OR). Phorbol myristate acetate (PMA; Sigma-Aldrich), ionomycin (Sigma-Aldrich), and the Cytofix/Cytoperm kit (BD Biosciences) were used in the preparation of cells for intracellular staining. CD4 and γδ T cells were purified using negative selection kits and CD8 T cells were purified using a positive selection kit (Miltenyi biotech, Auburn, CA) to study the cross-protective functions of different T cell subsets.

### CFSE-Based Flow Cytometric Assay to Study the Expansion of NTM Cross-Reactive T Cells

PBMC were labeled with CFSE (Molecular Probes) as recommended by the manufacturer. CFSE-labeled PBMC (1 × 10^6^/ml) were stimulated with live mycobacteria or the whole lysates mentioned above for 7 days at 37°C. Ranges of antigen concentrations were tested on samples from two volunteers and concentrations which were found to be optimal were used in all experiments. On day 7, the cells were restimulated with PMA (50 ng/ml) and ionomycin (750 ng/ml) in the presence of GolgiStop (0.7 μl/ml) for 2 h and studied for intracellular IFN-γ or GZM-A expression. Flow cytometric acquisition was performed on a multicolor BD FACS Canto II instrument, and analyses were done using FlowJo (Tree Star) software. A minimum of 10,000 events were acquired. Lymphocyte population was identified based on forward and side scatter. Then, CD3^+^ CD4^+^, CD3^+^ CD8^+^ and CD3^+^ γδ-TCR^+^ T cells were regated, and the CFSE low (CFSE^lo^) proliferating populations positive for IFN-γ and/or GZM-A were identified as effector subsets. The absolute numbers of effector populations were calculated by multiplying the percentage of each subset obtained with flow cytometry by the trypan blue-determined total viable cell counts. Stimulation indices (SI) were calculated by dividing the absolute numbers of T cell subsets after antigen expansion by the corresponding absolute numbers in controls rested in medium.

### Measurement of Th1/Th2/Th17 Cytokines in Culture Supernatants

PBMC from BCG-vaccinated or LTBI individuals were stimulated with BCG or rested in medium for 7 days. On day 7, cells were washed, pelleted, counted, and they were added to wells of 96-well plates containing MAV or MAB-infected autologous macrophages at an effector to target (E:T) ratio of 10:1. On day 10, cytokines in cocultures were measured using cytokine Bead Array (CBA), a multiplex assay allowing simultaneous detection of IL-2, IL-4, IL-6, IL-10, IL-17, TNF-α, and IFN-γ (BD Bioscience), was used to quantify levels of secreted cytokines in supernatants from co-cultures of MAV- and MAB-infected monocytes and T cells. The assays were performed according to the manufacturer's instructions and the data were analyzed using FCAP Array v 3.0 (BD) and a BD Cytometric Bead Array (BD Biosciences) software, in a FACS Canto TM flow cytometer (BD).

### Testing the Effects of BCG Vaccination and Mtb-Infection on NTM Cross-Reactive Immunity in Mice

Three groups of 8 week old C57BL/6 mice (The Jackson laboratories, Bar Harbor, ME) were used. The first group (*n* = 4) was kept without vaccination. The second group (*n* = 4) was vaccinated with BCG (10 × 10^6^) delivered intranasally (IN) under ketamine/xylazine induced anesthesia. The third group (*n* = 5) was vaccinated with BCG twice 4 weeks apart. Intranasal BCG vaccination was shown to be more immunogenic and protective compared to subcutaneous vaccination (24). Mice were euthanized 4 weeks after the last vaccination for studies of cross reactive mycobacterial specific immunity. Splenocytes (5 × 10^5^ cells/well) were stimulated overnight with live BCG or MAV at a multiplicity of infection (MOI) of 3, MAV-WL (10 μg/ml) or rested in medium. Frequencies of antigen-specific T cells were measured by ELISPOT assay (25). The results are presented as means ± SEs of IFN-γ spot forming cells (SFC) per million splenocytes. In similar experiments, C57BL/6 mice (*n* = 5) were administered a high-dose aerosolized Mtb challenge (Erdman strain; 5.5 × 10^7^CFU/ml) using a Glas Col Inhalation Exposure System (IES), resulting in a seeding of 1,700 Mtb CFU. Mice that did not receive aerosolized Mtb were used as uninfected controls. Mice were euthanized 4 weeks after infection, and IFN-γ ELISPOT assays were performed as described above after overnight stimulation of splenic cells with MAV-WL or MAB-WL (20 μg/ml).

### Testing the Effects of Recent BCG Vaccination on Cross-Reactive NTM Immunity in Humans

PBMC harvested pre- and 43-days post-BCG vaccination from five volunteers were labeled with CFSE as described above. CFSE labeled PBMC were stimulated with BCG at an MOI of 0.3, MAV-WL at a concentration of 2 μg/ml or MAB-WL at a concentration of 10 μg/ml for 7 days. On day 7, cells were stained for surface (CD3, CD4, CD8, and γδ-TCR) and intracellular (IFN-γ and granzyme A) markers. Flow cytometric acquisition and calculations of the absolute numbers of NTM cross-reactive T cells were performed as described above.

### Measuring the Capacity of Cross-Reactive T Cells to Inhibit Intracellular MAV and MAB

Mycobacterial growth inhibition assays were performed as described previously (26). First, we tested various mycobacterial concentrations for monocyte infection and the duration of the infection period before selecting a single MOI and time period of infection for further experiments. Briefly, adherent monocytes were infected for 4 h with MAB at an MOI of 1, overnight with MAV at an MOI of 3, or overnight with BCG at an MOI of 3. Extracellular mycobacteria were removed by washing 3 times. T cells expanded with BCG for 7 days were added to achieve an E:T ratio of 10:1, and co-cultures incubated at 37°C with 5% CO_2_ for 72 h. Cells were lysed with 0.2% saponin in RPMI 1640 medium, and the viable BCG bacilli released were quantified by CFU plating and/or [^3^H]uridine (GE Healthcare) incorporation. The percentages of mycobacterial growth inhibition were determined using the following formula: % inhibition = 100 – [100 × (CFU or counts per minute (CPM) in the presence of BCG-stimulated T cells/CFU or CPM in the presence of medium-rested T cells)]. CPM was used for slow growing mycobacteria (BCG and MAV) and CFU was used for MAB.

## Results

### T Cells From PPD-Positive Individuals Are Reactive to NTM Stimulation

**Figure 1:**
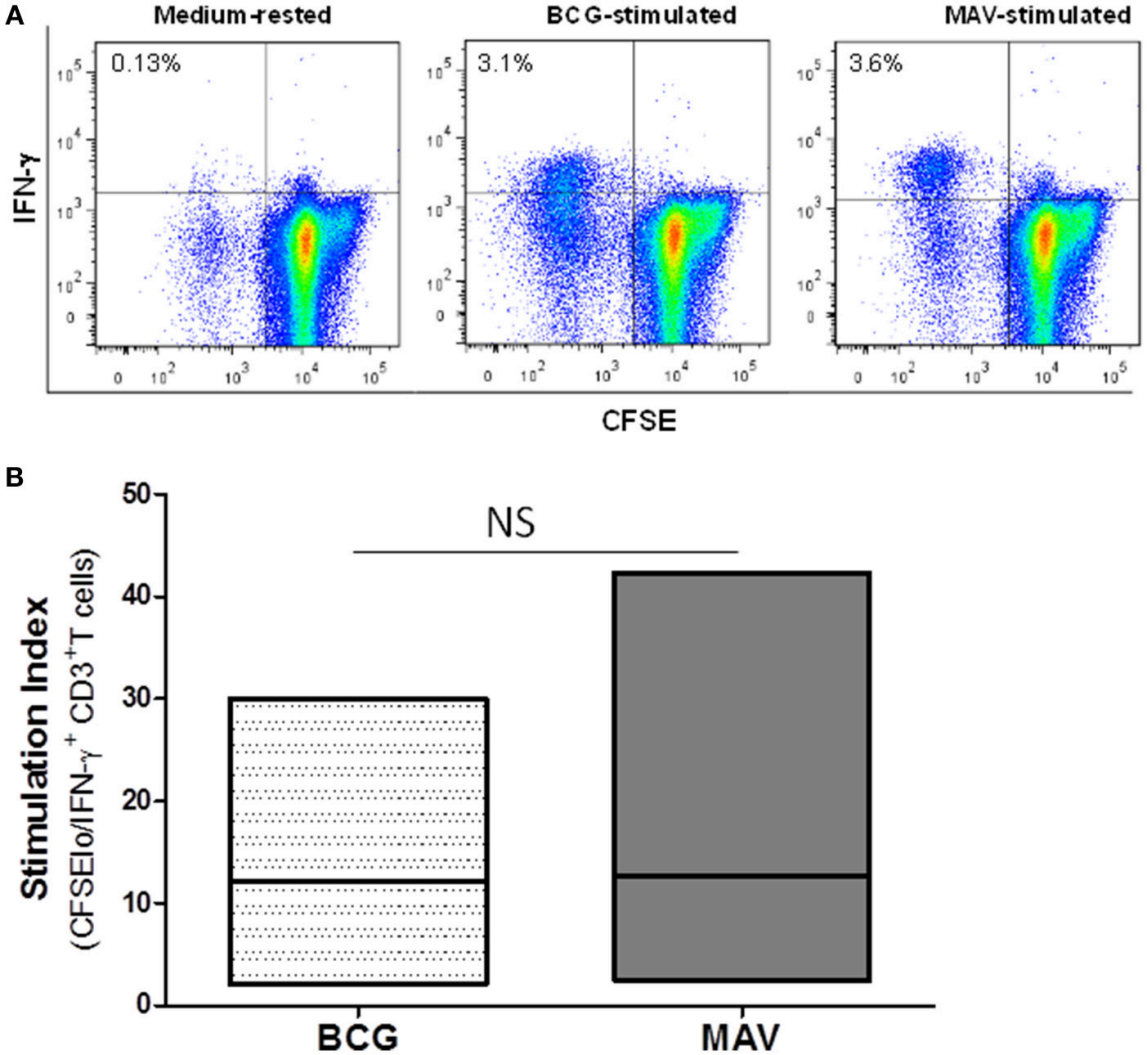
Previous exposure to BCG or Mtb induces cross-reactive T cells against NTM. PBMC from BCG-vaccinated and/or latent TB infected individuals (*n* = 6) were CFSE-labeled and stimulated with optimal concentrations of live BCG (Connaught) or MAV (ATCC 700898). On day 7, cells were restimulated with PMA/ionomycin and the total percentages of CFSE^lo^ (proliferating) and IFN-γ producing T cells were determined by flow cytometry. **(A)** Flow cytometry plots of a single volunteer. Lymphocytes gated on the basis of forward and side scatter and then regated on CD3^+^ cells were analyzed for CFSE and IFN-γ expression by use of FlowJo software. The number in the upper left quadrant of each dot plot refers to the percentage of CFSE^lo^IFN-γ^+^CD3^+^T cells detected after *in vitro* stimulation. **(B)** Composite data from 6 volunteers. Stimulation indices were calculated by dividing the absolute numbers of CFSE^lo^IFN-γ^+^CD3^+^ T cells in cultures containing BCG or MAV by the absolute numbers of CFSE^lo^/IFN-γ^+^ CD3^+^T cells in medium-rested cultures. Stimulation with MAV led to expansion of effector T cells comparable to the level obtained with BCG stimulation (*P* > 0.05, Wilcoxon matched-pairs test). The bars show ranges and the lines show mean values. NS, not significant.

**Figure 2:**
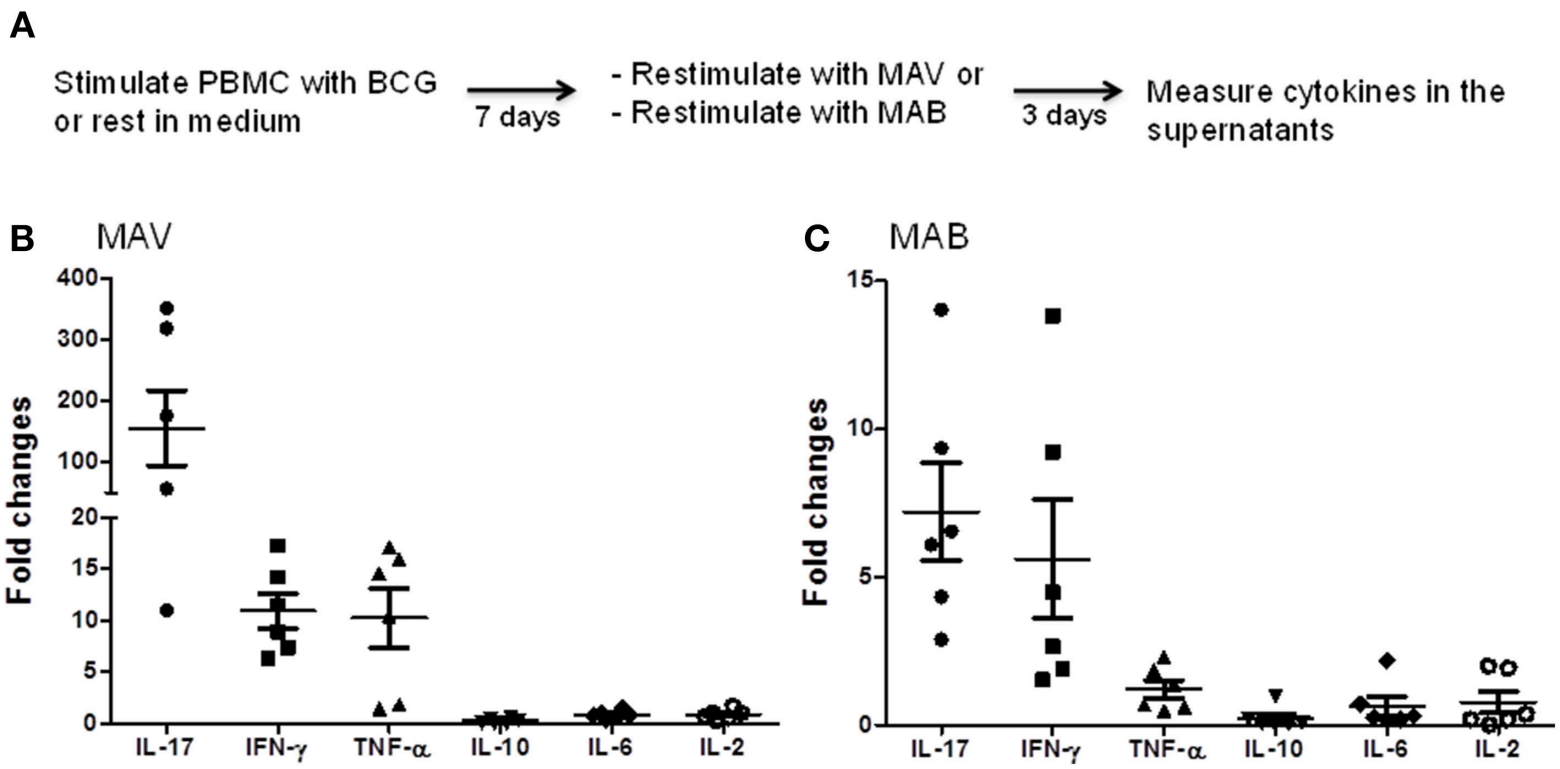
MAV and MAB cross-reactive immunity includes Th1 and Th17 responses. **(A)** Schematic of experiments conducted to measure MAV or MAB cross-reactive immunity. PBMC from BCG-vaccinated or latent TB infected individuals (*n* = 6) were stimulated with BCG or rested in media for 7 days. Then, these expanded cells were co-cultured with MAV- or MAB-infected autologous monocytes at an E:T ratio of 10:1. On day 3 of co-culture, Th1, Th2, and Th17 responses were measured in co-culture supernatants using CBA. Fold changes for each cytokine was calculated by dividing the amount of cytokine produced following restimulation with MAV or MAB by the amount produced in medium-rested cultures. **(B)** Exposure of BCG-expanded T cells to MAV-infected macrophages increased IL-17, IFN-γ, and TNF-α by 156 ± 62, 11 ± 1.7, 10.3 ± 2.9 pg/ml (Mean ± SE), respectively. IL-10, IL-6, and IL-2 showed no marked changes with fold changes (mean ± SE) of 0.3 ± 0.1, 0.9 ± 0.2, 0.9 ± 0.2 pg/ml, respectively. **(C)** Similarly, exposure of BCG-expanded T cells to MAB-infected macrophages increased IL-17 and IFN-γ in by 7.2 ± 1.6, and 5.6 ± 2, mean fold ± SE. There were no marked changes in the levels of TNF-α, IL-10, IL-6, and IL-2 with fold changes (mean ± SE) of 1.2 ± 0.3, 0.2 ± 0.2, 0.6 ± 0.3, 0.8 ± 0.4 pg/ml, respectively.

To study the effects of prior exposure to TB or BCG on NTM immunity, we performed a CFSE-based flow cytometric assay which enables the identification of T cells that proliferate (become CFSE low) and produce effector molecules in response to antigen-specific stimuli (27–29). Briefly, PBMC from PPD-positive subjects were labeled with CFSE and stimulated with BCG, MAV or MAB. On day 7, the proportions of T cells that proliferated and expressed IFN-γ or GZM-A were determined by flow cytometry. Figure 1 shows that Mtb immunity, following BCG vaccination or latent TB infection, induces NTM cross-reactive T cells. The responses to MAV were comparable with the responses to BCG stimulation indicating the abundance of NTM cross-reactive T cells among BCG and Mtb-specific T cells. The SIs (mean ± SE) for total CFSE^lo^IFN-γ^+^CD3^+^ responses were 12.2 ± 4.7 and 12.7± 7.5 after stimulation with BCG and MAV, respectively. These responses to MAV were not markedly different from the response to BCG (*P* = 0.5, Wilcoxon matched-pairs test, *n* = 5). Experiments to compare the expansion with BCG, MAV-WL, MAB-WL and Mtb-PPD showed similar results (Supplement Figure 1). To confirm the presence of NTM cross-reactive T cells among BCG-specific T cells, we co-cultured BCG-expanded T cells with MAV- or MAB-infected autologous macrophages for 3 days and measured Th1/Th2/Th17 cytokines in culture supernatants using CBA. The experimental outline is shown in Figure 2A. Figures 2B,C show fold changes (mean ± SE) of cytokines following restimulation with MAV and MAB compared to medium-rested T cells. The cytokine concentration in the different cocultures is shown in Supplement Figure 2. In control cultures containing BCG-expanded T cells, there was no measurable IL-2 and Il-10 but IL17, IFN-γ, TNF-α, and IL-6 concentrations (mean pg/ml ± SE) were 11.3 ± 10.5, 213 ± 80, 188 ± 9.8, 231 ± 39, respectively.Exposure of BCG-expanded T cells to MAV-infected macrophages increased the production of IL17, TNF-α, and IFN-γ by 156 ± 62, 11 ± 1.7, 10.3 ± 2.9 pg/ml (Mean ± SE), respectively. Similarly, exposure of BCG-expanded T cells to MAB-infected macrophages increased IL-17 and IFN-γ in by 7.2 ± 1.6, and 5.6 ± 2 pg/ml. These results do not address the possibility that the PPD-positivity of volunteers or *in vitro* T cell responses could be from prior exposure to NTMs. Therefore, in subsequent experiments, in addition to further characterizing NTM cross-reactive T cells, we performed well-controlled murine and human experiments to avoid interference of prior NTM infection on cross-reactive T responses.

### NTM Cross-Reactive T Cells From PPD+ Individuals Are Capable of Inhibiting Intracellular NTM Replication

**Figure 3:**
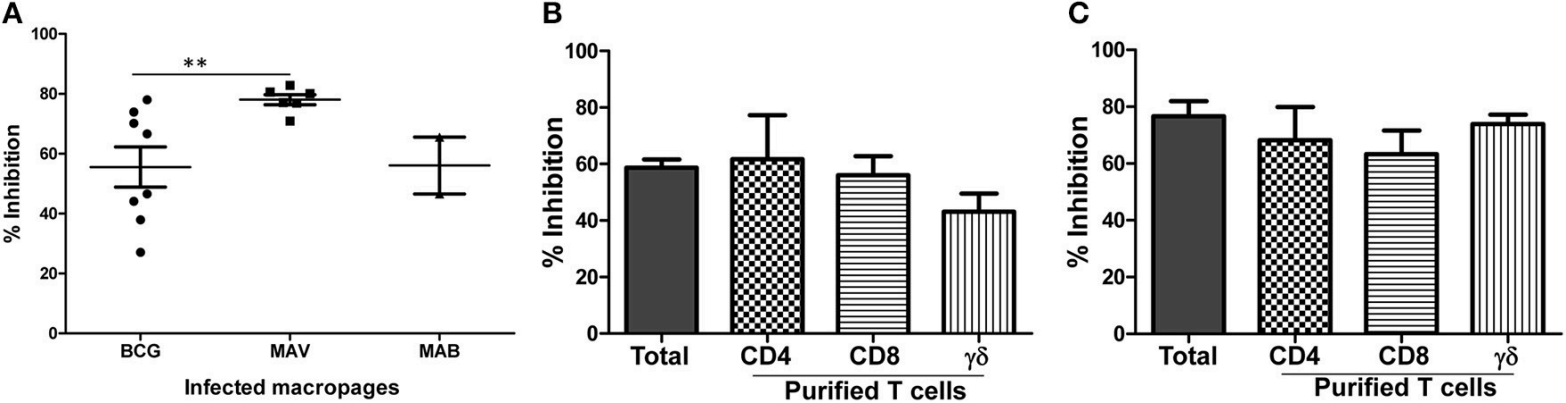
BCG-specific T cells cross-protect against MAV and MAB. Total PBMC or subsets of T cells purified after 7 days of optimal BCG stimulation were co-cultured with autologous macrophages infected with MAV or MAB (E:T of 10). Residual mycobacteria quantified 3 days after co-culture and percent inhibition calculated by dividing the number of residual mycobacteria in the presence BCG-stimulated PBMC by the number of residual mycobacteria in control cultures containing medium-rested PBMC. **(A)** BCG-expanded T cells inhibit intracellular MAV (*n* = 8) and MAB (*n* = 5) as potently as they inhibit intracellular BCG (*n* = 8). BCG-expanded T cells inhibited intracellular MAV better than intracellular BCG (**p < 0.01, Mann-Whitney U test). **(B)** Pure CD4, CD8, and γδ T cells inhibited intracellular MAV, and the level of inhibition was similar to inhibition by total BCG-expanded PBMC. **(C)** Pure CD4, CD8, and γδ T cells inhibited intracellular MAB, and the level of inhibition is similar to the levels of inhibition mediated by total BCG-expanded PBMC.

To further elucidate the function of NTM cross-reactive T cells, we adopted our *in vitro* protection assay, in use for several years for studies of TB-specific immunity, for studies of MAV and MAB intracellular replication (26, 30–32). In order for this assay to work effectively, NTM need to replicate intracellularly within human macrophages. Therefore, we first studied the growth kinetics of MAV and MAB inside human macrophages. Supplement Figures 3A,B demonstrate that both MAV and MAB replicate efficiently inside human macrophages. Next, we tested the capacity of cross-reactive T cells to inhibit intracellular NTM growth by co-culturing infected macrophages with *in vitro* expanded BCG-specific T cells as described in Methods. Figure 3A shows that BCG-expanded T cells inhibit intracellular MAV and MAB. The levels of inhibition of intracellular MAV or MAB by BCG-expanded T cells were similar to or better than the levels of intracellular BCG inhibition. These results indicate the potential of BCG-expanded NTM cross-reactive T cells to protect against NTMs.

### CD4, CD8 and γδ T Cell Subsets Induced by BCG Vaccination Inhibit Intracellular NTM

We next identified the different subsets of T cell to inhibit intracellular MAV and MAB. Briefly, we purified CD4, CD8, and γδ T cell subsets from BCG-expanded PBMC using immunomagnetic cell selection kits (Miltenyi biotech). Purified CD4, CD8, and γδ T cells were co-cultured with MAV or MAB-infected autologous macrophages as described above. Typical flow cytometric plot of the purity of subsets of T cells used in these experiments is shown in Supplement Figure 4. CPM and CFU values for MAV and MAB, respectively are shown in Supplement Figure 5. Percent inhibition was calculated to determine the relative abilities of each T cell subset to inhibit intracellular MAC and MAB. Figure 3B shows that CD4, CD8, and γδ T cells efficiently inhibited intracellular MAV with percent inhibitions of 62% ± 15.6, 56% ± 6.7, and 42% ± 6.4, respectively. Furthermore, Figure 3C demonstrates that CD4, CD8, and γδ T cells also inhibited intracellular MAB with percent inhibitions of 68% ± 11.7, 63% ± 8.3 and 74% ± 3.3, respectively. These results further confirm that CD4, CD8, and γδ T cells induced by BCG are important for cross-protection against MAV and MAB.

### BCG Vaccination Expands NTM Cross-Reactive Immunity

**Figure 4:**
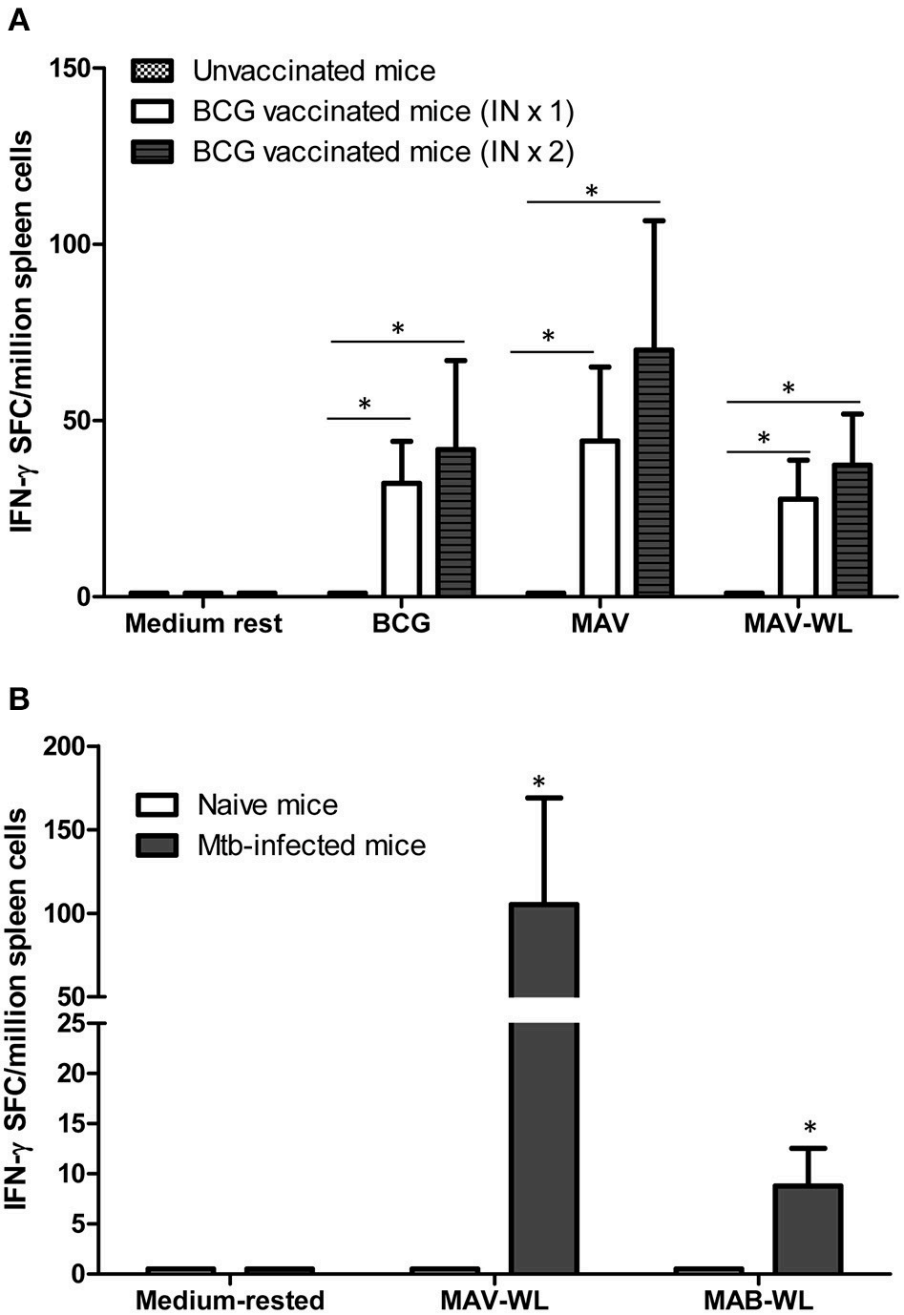
BCG vaccination or Mtb infection of mice induces MAV and MAB reactive immunity. **(A)** Groups of C57BL/6 mice (*n* = 4–5 per group) were vaccinated once or twice intranasally with BCG. Four weeks later mice were euthanized. Splenic cells were harvested from vaccinated and control mice. Cells were rested in medium or stimulated overnight with live BCG at MOI of 3, MAV at MOI of 3, and MAV-WL in IFN-γ ELISPOT assays. Shown are the means ± SE of IFN-γ SFC per million splenic cells. The number of IFN-γ SFC following stimulation with BCG, MAV, and MAV-WL were significantly higher in mice which received one or two BCG vaccinations compared to unvaccinated mice (*P < 0.05, Mann-Whitney U test). The number of mycobacteria-induced IFN-γ SFC were similar following one versus two BCG vaccination (*P* > 0.05). **(B)** C57BL6 infected with aerosolized Mtb (*n* = 5) were euthanized 4 weeks after infection. Splenic cells from uninfected and infected mice were used in IFN-γ ELISPOT assays. Mtb-infected mice had significantly more MAV and MAB cross-reactive IFN-γ SFC compared to uninfected mice (*P < 0.05, Mann-Whitney U test).

To better understand changes in NTM immunity after BCG vaccination and Mtb infection, we vaccinated mice with one or two-doses of BCG, 4 weeks apart. Figure 4A demonstrates that BCG-induced MAV cross-reactive immunity assessed by IFN-γ ELISPOT assays detectable in splenic cells following *in vitro* stimulation with BCG or MAV antigens. Mice vaccinated with one or two doses of BCG had significantly higher numbers of MAV cross-reactive splenic cells compared to unvaccinated controls (*P* < 0.05). Similarly, Mtb infected mice had higher numbers of MAV and MAB cross-reactive splenic cells compared to uninfected controls (*P* < 0.05, Figure 4B).

**Figure 5:**
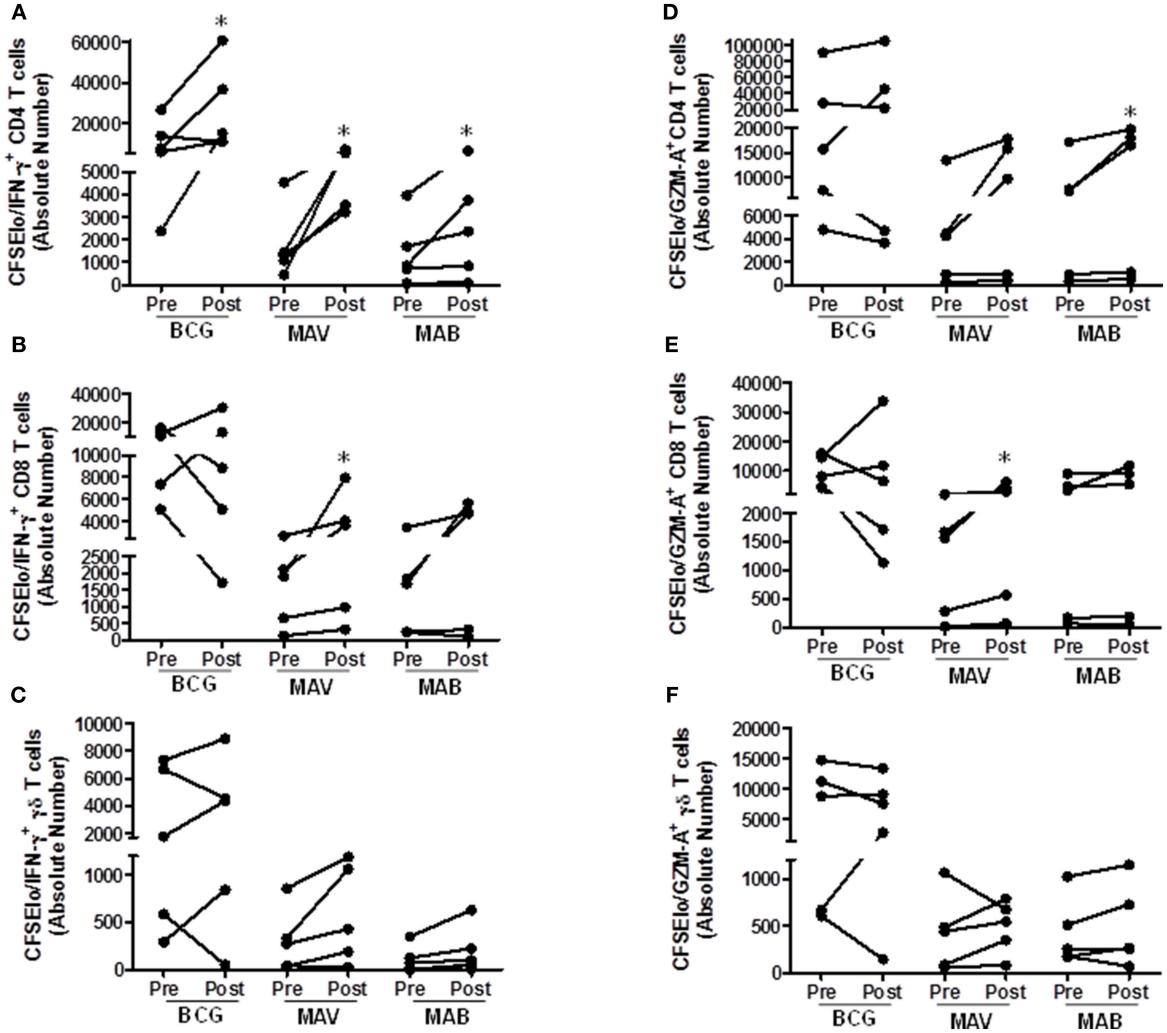
BCG vaccination in humans induces MAV and MAB cross-reactive T cells. Paired pre-and post-vaccination PBMC from recently BCG vaccinated volunteers living in St. Louis, MO (*n* = 5) were used. PBMC were labeled with CFSE and stimulated with BCG, MAB WL, or MAB WL. Medium rested PBMC were used as negative controls. On day 7, cells were restimulated with PMA/ionomycin for 2 h, viable cells were counted and cells were stained for surface and intracellular markers for flow cytometry study. **(A–C)** show the data for proliferating and IFN-γ producing T cells. **(D–F)** show the data for proliferating and GZM-A producing T cells. There were significantly higher absolute numbers (AN, per ml of cultures) of BCG-reactive CFSE^lo^IFN-γ^+^CD4^+^ T cells (*P* = 0.03, Wilcoxon Matched Pairs test), MAV WL reactive CFSE^lo^IFN-γ^+^CD4^+^ T cells (*P* = 0.03), MAV WL reactive CFSE^lo^IFN-γ^+^CD8^+^ T cells (*P* = 0.03), MAV WL reactive CFSE^lo^Granzyme A^+^CD8^+^ T cells (*P* = 0.03), MAB WL reactive CFSE^lo^IFN-γ^+^CD4^+^ T cells (*P* = 0.03), and MAB WL reactive CFSE^lo^Granzyme A^+^CD4^+^ T cells (*P* = 0.03), indicating that BCG induces NTM cross-reactive immunity. *P < 0.05.

We further tested the effects of BCG administration on NTM T cell immunity in humans. For this purpose, we used pre- and post-vaccination PBMC from recently BCG-immunized volunteers living in the U.S. The volunteers had no prior history of BCG vaccination and had no history of travel to high TB endemic countries. Figure 5 shows that these individuals had some baseline T cell responses to both MAV and MAB. However, these responses were significantly higher in post-BCG vaccination samples. The total proliferating cells in medium rested PBMC and PBMC stimulated with the different antigens are shown in Supplement Figure 6. Typical gating of CFSE and other markers are shown in Supplement Figure 7. Figure 5 shows that absolute numbers/ml of culture (median/IQR) of MAV-responsive CFSE^lo^IFN-γ^+^ CD4^+^ T cells increased from 1,321/2,214 in pre-vaccination PBMC to 5,379/3,480 in post-vaccination PBMC (*P* = 0.03, Wilcoxon-matched pairs test, *n* = 5). Similarly, CFSE^lo^IFN-γ^+^ CD8^+^ T cells increased from 1,909/2,002 to 3,647/5,361 (P = 0.03, Wilcoxon-match pairs test, *n* = 5), and CFSE^lo^IFN-γ^+^γδ TCR^+^ T cells increased from 272/556 to 428/1,018 (*P* = 0.062, Wilcoxon-matched pairs test, *n* = 5). In addition, absolute numbers of MAV cross-reactive CFSE^lo^GZM-A^+^CD8^+^ T cells increased from 1,555/1712 to 3,068/4910 (*P* = 0.03, Wilcoxon matched-pairs test), absolute numbers of MAB cross-reactive CFSE^lo^IFN-γ^+^CD4^+^T cells increased from 853/1247 to 2,367/3118 and CFSE^lo^GZM-A^+^CD4^+^T cells increased from 7,235/11758 to 16,449/18,050 (*P* = 0.03, Wilcoxon matched-pairs test).

## Discussion

Epidemiological evidences suggest that BCG vaccination and latent TB infection decrease the risk of developing NTM disease but the exact mechanisms have not been studied (20, 21). Because cross-protective immunity seems to be a plausible explanation, we carried out robust immunological tests. Previous studies have shown that pathogen effector T cells proliferate and produce effector molecules such as IFN-γ (28, 29). We have used similar assays which measure proliferation and expression of effector molecules in our vaccine studies before (33, 34). We selected MAV and MAB for these studies since they are the most common causes of pulmonary NTM in North America (12) and demonstrated, for the first time, that both BCG and Mtb infection induce MAV and MAB cross-reactive T cells. In most of our assays, live NTM was used when quantification of residual mycobacteria was required, and whole lysates of NTM alone or in combination with live NTM were used for flow cytometric assays.

In humans, it is widely known that HIV/AIDS patients with low CD4 counts have a high risk of developing disseminated MAC, indicating the importance of CD4 T cells in NTM immunity (35). In HIV-negative patients, pulmonary NTM is the most common form of NTM disease (36). HIV-negative patients without predisposing lung disease who develop pulmonary MAC have increased numbers of CD4 and CD8 T cells as well as higher levels of TNF-α, IL-1β, IL-6, and IL-8 in bronchoalveloar fluid (BAL), suggesting the possibility that both CD4 and CD8 T cells contribute to MAC immunity (37). In fact, the CD4 T cell number in lung air way cells decreases as the bacterial load increases, likely due to apoptosis (38–40). A similar finding was obtained with MAB; increased bacterial load causes a decrease in the number of MAB specific T cells (41). This could be the main reason why patients with advanced pulmonary MAC or MAB have a decreased Th1 response with low levels of IFN-γ (42–44) which is reversible with effective antibiotic treatment (45, 46). Unfortunately, pulmonary NTM is difficult to manage with high failure rates, and the emergence of drug-resistant NTMs have made management of these cases more challenging, if not impossible, indicating the need for vaccines and host-directed immunotherapies. Our study provides the first evidence that T cells expanded with BCG cross-react with MAV and MAB, with further increases in proliferating and IFN-γ expressing T cells as shown in CFSE-based assays or increased production of IFN-γ, TNF-α, and IL-17 as shown with CBA assays. These findings indicate that BCG induces cross-reactive NTM immunity similar to immune responses seen in patients with NTM during antibiotic therapy (37, 44). This is likely due to shared antigens between BCG, Mtb and clinically relevant NTM.

Because concomitant exposure to NTM may give false-positive results, we performed experiments in mice after BCG vaccination and Mtb infection. These experiments generated similar results, with increases in NTM cross-reactive splenic cell responses detected following BCG or Mtb infection. Previous studies in animals have shown that T cell immunity involving CD4 and CD8 T cells is important for protection against NTM disease. Intranasal infection of mice or intra-bronchial infection of non-human primates with MAC leads to activation of both CD4 and CD8 T cells (19, 47). Similarly, mice or guinea pigs infected with aerosolized MAB exhibit an early influx of IFN-γ+ T cells preceding successful clearance (48). Further studies have shown that depletion of CD4 T cells or neutralization of IFN-γ causes worsening of MAC and MAB infection in mice (49–51). Control of MAC and MAB infection also requires other key cytokines such as TNF-α and IL-12 (49, 52–54). Notably, TNF-α-deficient mice are unable to control MAC infection (53). Knock out of IL-12 in mice leads to a predictably marked decrease in the number of IFN-γ producing mycobacteria-specific T cells recruited to the lung and a significant increase in bacterial load (49, 54).

Like Mtb, MAC and MAB are intracellular mycobacteria which interfere with the functions of antigen presenting cells (APC). Both MAC and MAB inhibit phagosome-lysosome fusion and therefore, prevent their killing by APC (55, 56). As infection progresses, their unabated replication in professional APCs causes apoptosis, thereby facilitating further spreading (57–59). Interestingly, both MAC and MAB escape the phagosome during apoptosis and infect newly recruited uninfected APC making them niches for survival and further replication (58–60). Our results on intracellular growth kinetics confirm that both MAV and MAB replicate efficiently inside human macrophages. In the absence of effector T cells, the effects of key cytokines with autocrine activities such as TNF-α on macrophages are not sustainable as these organisms are able to reduce macrophage responsiveness and suppress signal transducers (61, 62). Thus, we optimized a functional assay to measure the effects of BCG-expanded T cells on the intracellular growth of MAV and MAB. These functional assays measuring the ability of effector T cells to inhibit the growth of intracellular mycobacteria have been used as biomarkers of potential protection in new TB vaccine trials (32, 34, 63). Our results demonstrated that BCG-expanded T cells inhibit intracellular MAV and MAB with the same potency as they inhibit intracellular BCG. Furthermore, BCG-expanded purified CD4, CD8, and γδ T cells exhibit potent MAV and MAB inhibitory activities, confirming that all of these 3 subsets of T cells are important for NTM immunity in humans. Our study has the following limitations: (1) clinical strains of NTM were not used and (2) samples from mice and humans with immunodeficiency and/or structural lung disease were not included although these are the most common risk factors for pulmonary NTM (6, 64). In conclusion, our findings indicate that BCG induces MAV and MAB cross-reactive T cells with the ability to inhibit intracellular replication. These findings highlight the potential of BCG and other new TB vaccines for cross-protective prophylactic and immunotherapeutic effect against clinically relevant NTM. Future studies should address the effects of cross-reactive T cells on clinical strains of NTM in models that include structural lung disease and/or immunodeficiency that are common in patients at high risk of developing pulmonary NTM. Models demonstrating the immune deficits in NTM patients will better provide information on which dysfunctional cell phenotypes require modulation to ultimately induce protective immunity by vaccination. In addition, the mechanisms of inhibition of intracellular NTM need to be studied further to have better understanding of the potential of BCG as NTM vaccine.

## Ethics Statement

The protocol for collection and use of human samples was approved by the Saint Louis University Institutional Review Board (IRB), and informed consent was obtained from each volunteer. Human research was carried out according to the principles of the Declaration of Helsinki.

Murine studies were approved by the Saint Louis University's institutional animal care and use committee and all the animal experiments were done in accordance with biosafety regulations of the Saint Louis University.

## Author Contributions

GA conceptualized the study. FH, CE, and GA performed the experiments. GA and DH supervised the study. GA and FH analyzed the data. GA wrote the manuscript with input from all authors. All authors approved the final manuscript.

### Conflict of Interest Statement

The authors declare that the research was conducted in the absence of any commercial or financial relationships that could be construed as a potential conflict of interest.
