# Supplementary material for: BCG Vaccination Induces M. avium and M. abscessus Cross-Protective Immunity
Source: Front Immunol. 2019 Feb 19;10:234. doi: 10.3389/fimmu.2019.00234 (PMC6389677; doi:10.3389/fimmu.2019.00234)
Supplement: Supplementary file 1 [file Presentation_1.PPTX]

## Slide 1
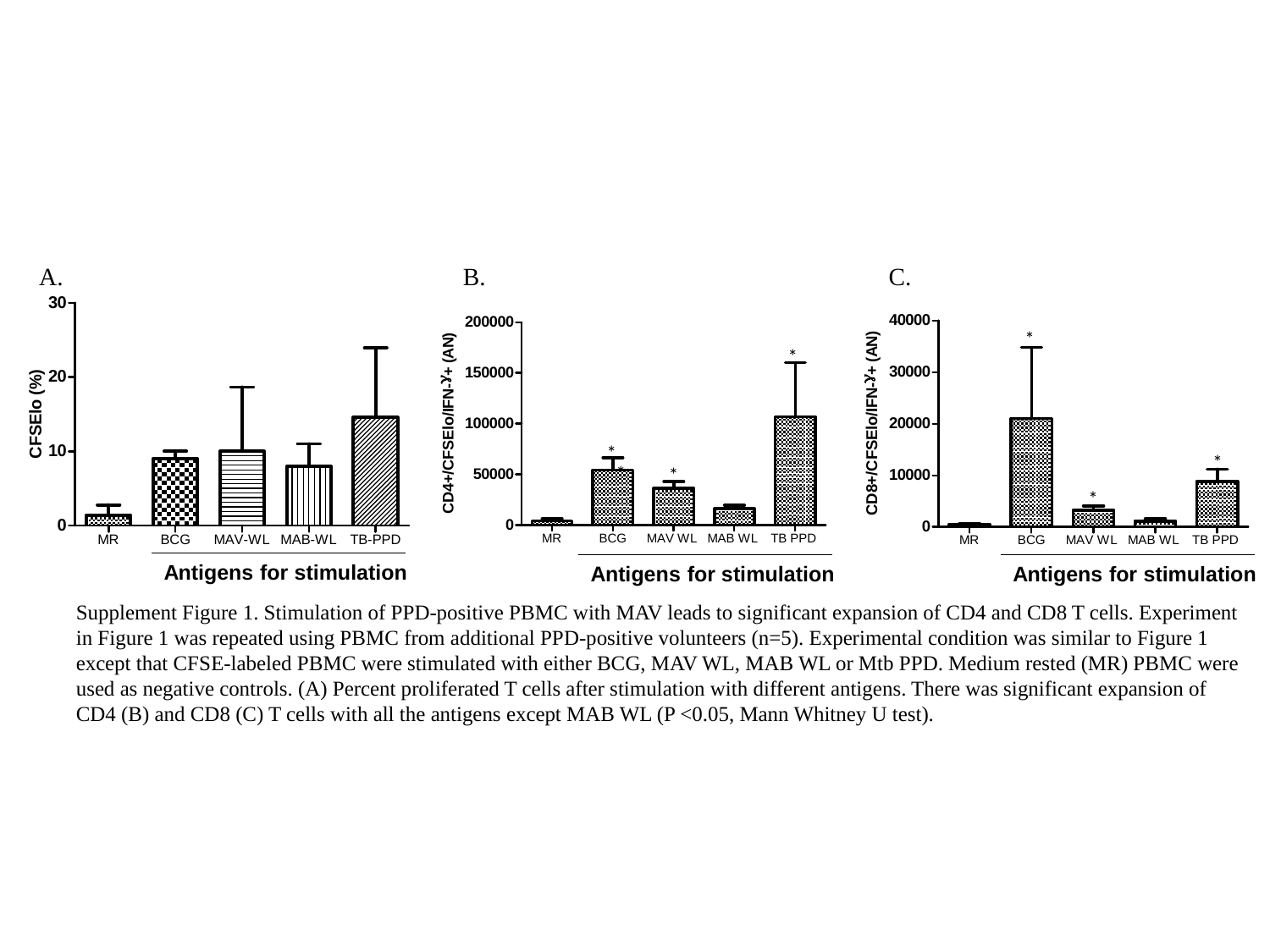

Supplement Figure 1. Stimulation of PPD-positive PBMC with MAV leads to significant expansion of CD4 and CD8 T cells. Experiment in Figure 1 was repeated using PBMC from additional PPD-positive volunteers (n=5). Experimental condition was similar to Figure 1 except that CFSE-labeled PBMC were stimulated with either BCG, MAV WL, MAB WL or Mtb PPD. Medium rested (MR) PBMC were used as negative controls. (A) Percent proliferated T cells after stimulation with different antigens. There was significant expansion of CD4 (B) and CD8 (C) T cells with all the antigens except MAB WL (P <0.05, Mann Whitney U test).

## Slide 2
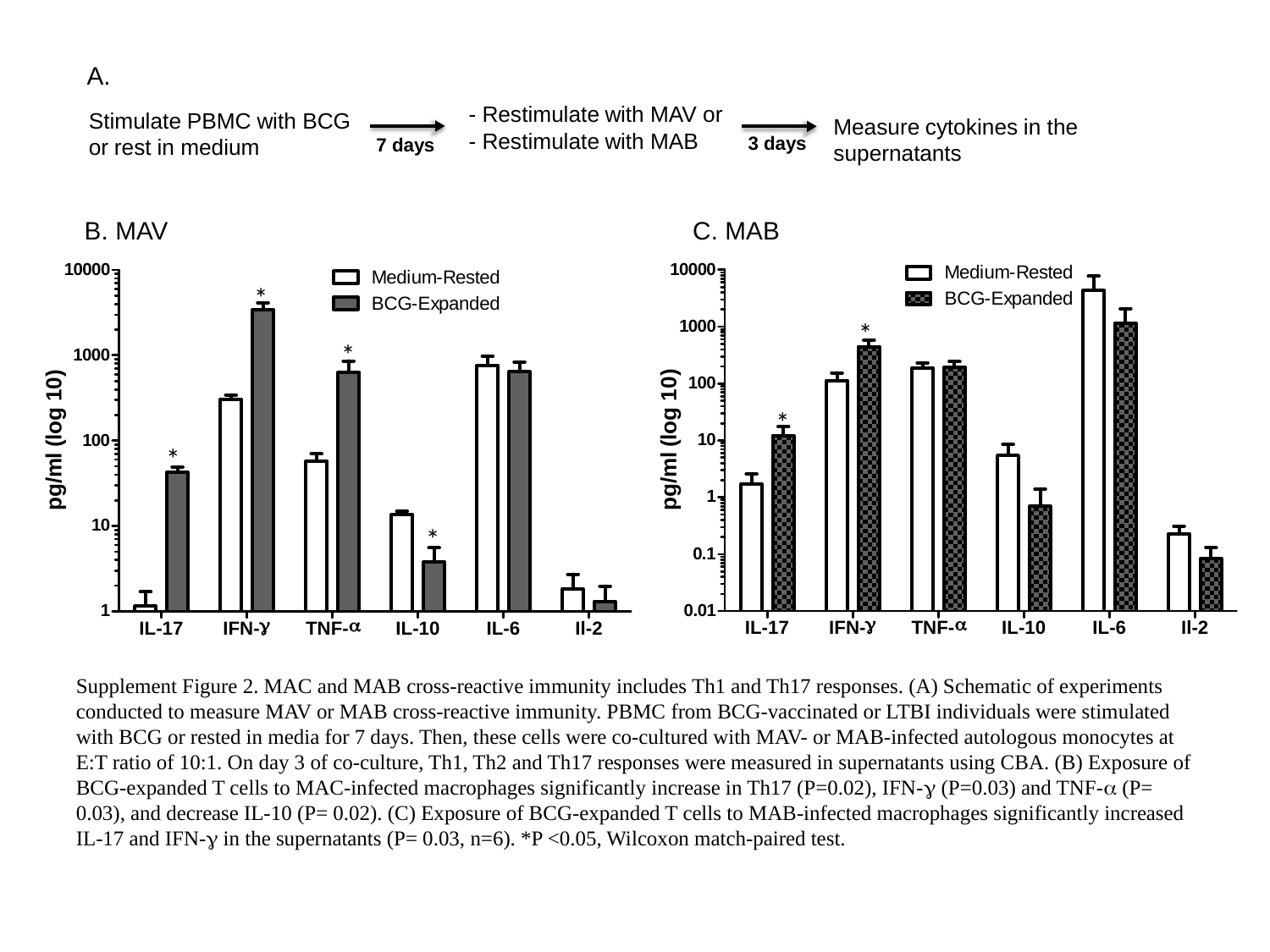

Supplement Figure 2. MAC and MAB cross-reactive immunity includes Th1 and Th17 responses. (A) Schematic of experiments conducted to measure MAV or MAB cross-reactive immunity. PBMC from BCG-vaccinated or LTBI individuals were stimulated with BCG or rested in media for 7 days. Then, these cells were co-cultured with MAV- or MAB-infected autologous monocytes at E:T ratio of 10:1. On day 3 of co-culture, Th1, Th2 and Th17 responses were measured in supernatants using CBA. (B) Exposure of BCG-expanded T cells to MAC-infected macrophages significantly increase in Th17 (P=0.02), IFN-γ (P=0.03) and TNF-α (P= 0.03), and decrease IL-10 (P= 0.02). (C) Exposure of BCG-expanded T cells to MAB-infected macrophages significantly increased IL-17 and IFN-γ in the supernatants (P= 0.03, n=6). *P <0.05, Wilcoxon match-paired test.

## Slide 3
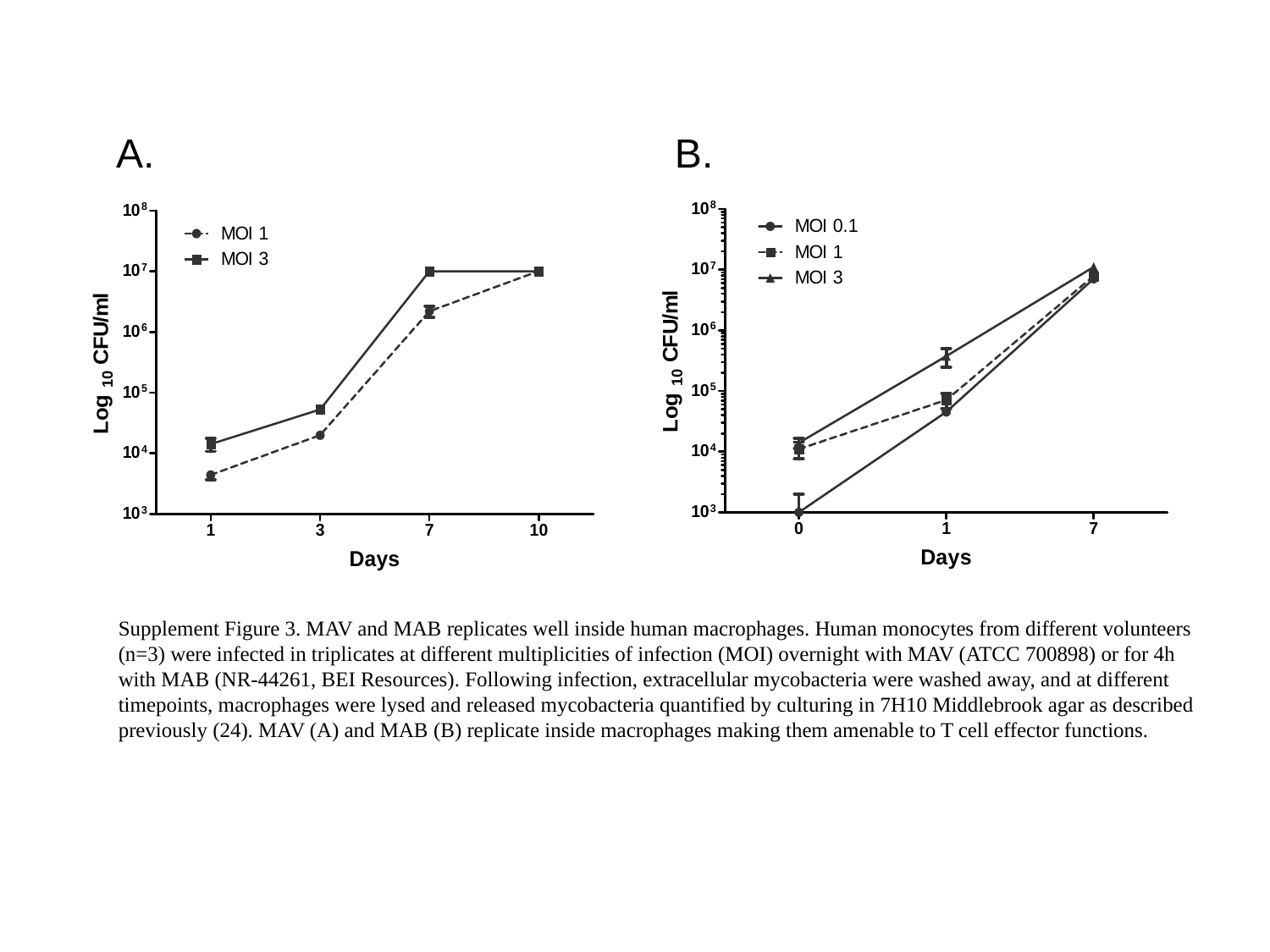

Supplement Figure 3. MAV and MAB replicates well inside human macrophages. Human monocytes from different volunteers (n=3) were infected in triplicates at different multiplicities of infection (MOI) overnight with MAV (ATCC 700898) or for 4h with MAB (NR-44261, BEI Resources). Following infection, extracellular mycobacteria were washed away, and at different timepoints, macrophages were lysed and released mycobacteria quantified by culturing in 7H10 Middlebrook agar as described previously (24). MAV (A) and MAB (B) replicate inside macrophages making them amenable to T cell effector functions.

## Slide 4
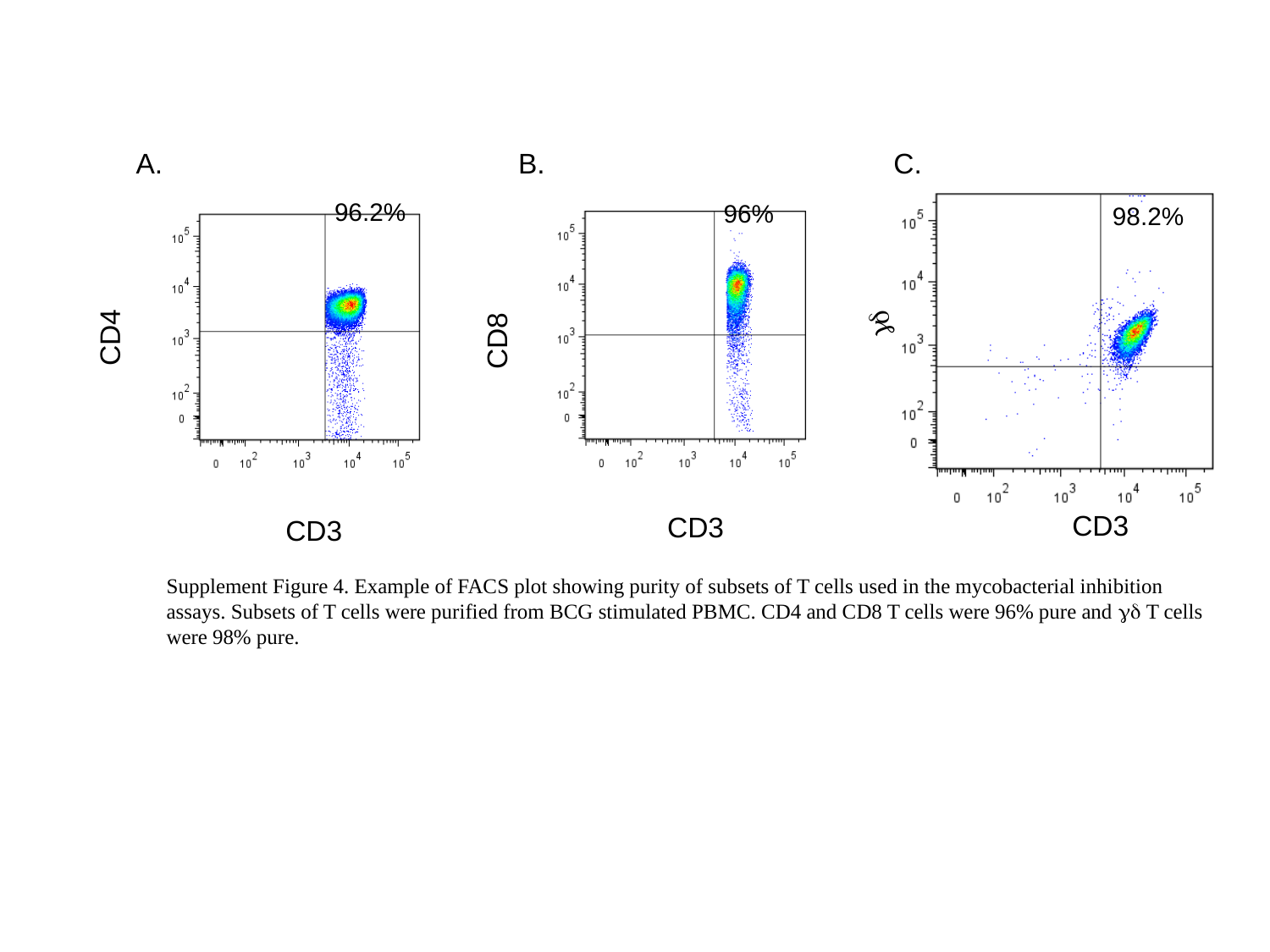

Supplement Figure 4. Example of FACS plot showing purity of subsets of T cells used in the mycobacterial inhibition assays. Subsets of T cells were purified from BCG stimulated PBMC. CD4 and CD8 T cells were 96% pure and  T cells were 98% pure.

## Slide 5
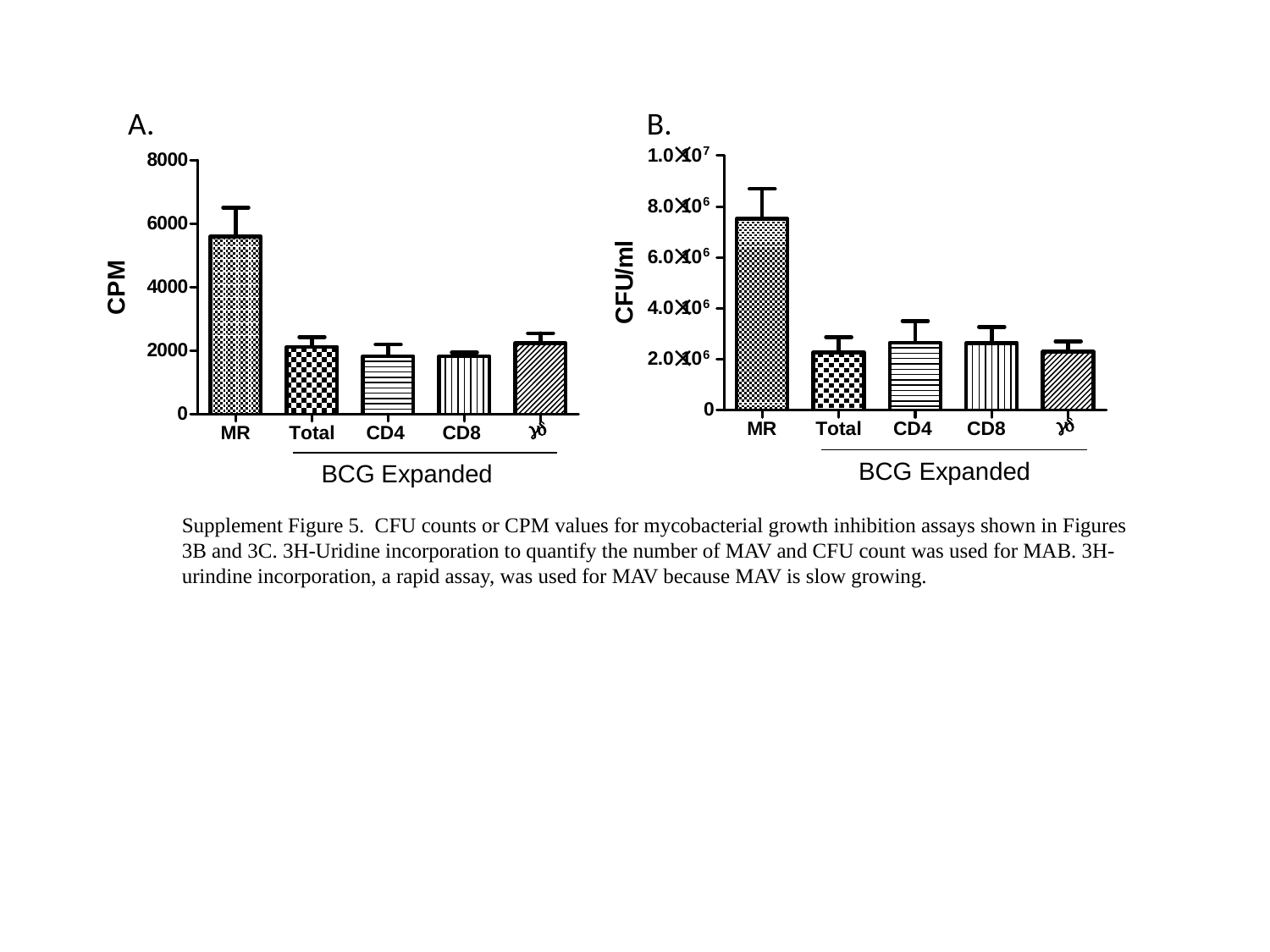

Supplement Figure 5. CFU counts or CPM values for mycobacterial growth inhibition assays shown in Figures 3B and 3C. 3H-Uridine incorporation to quantify the number of MAV and CFU count was used for MAB. 3H-urindine incorporation, a rapid assay, was used for MAV because MAV is slow growing.

## Slide 6
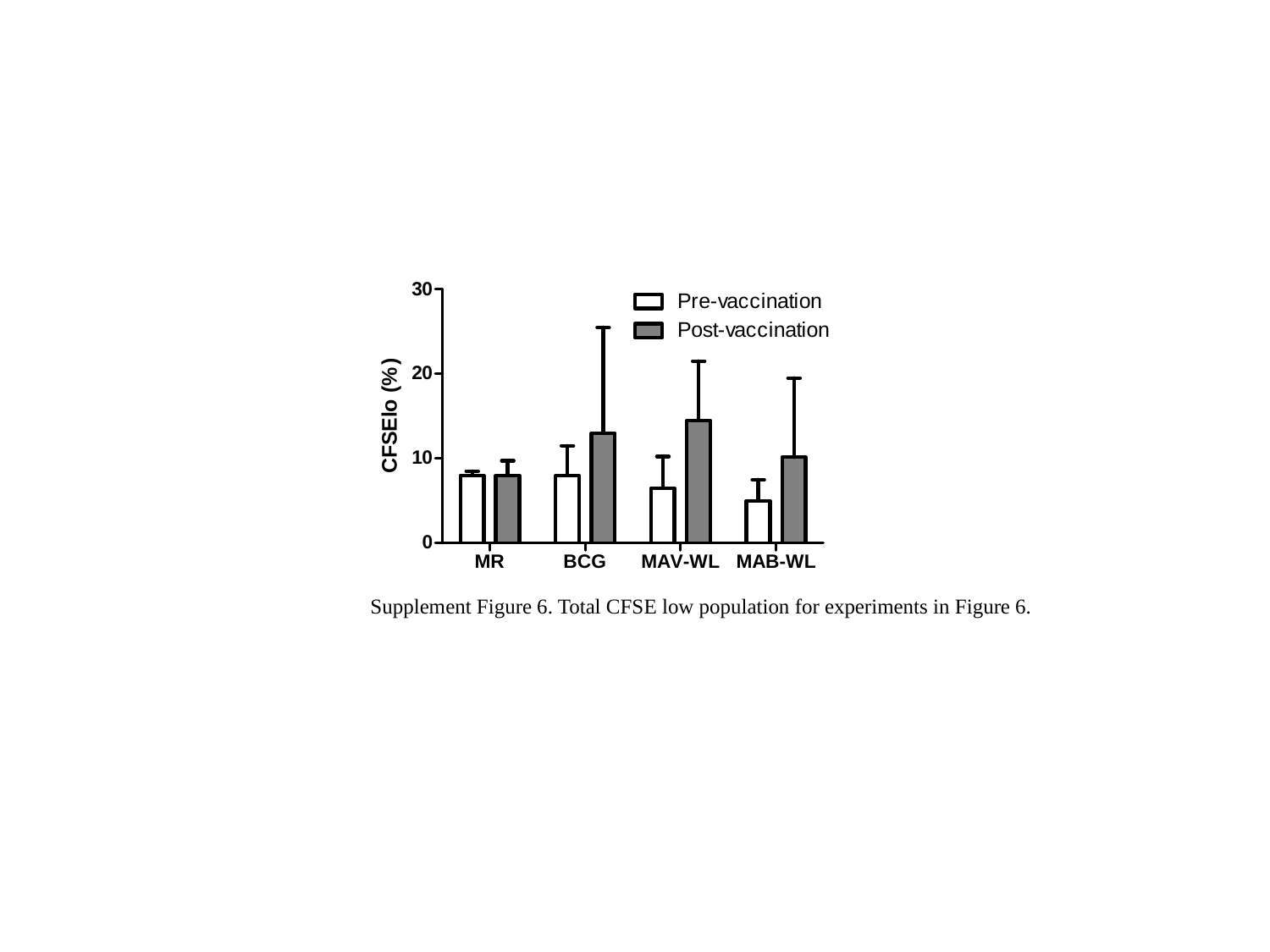

Supplement Figure 6. Total CFSE low population for experiments in Figure 6.

## Slide 7
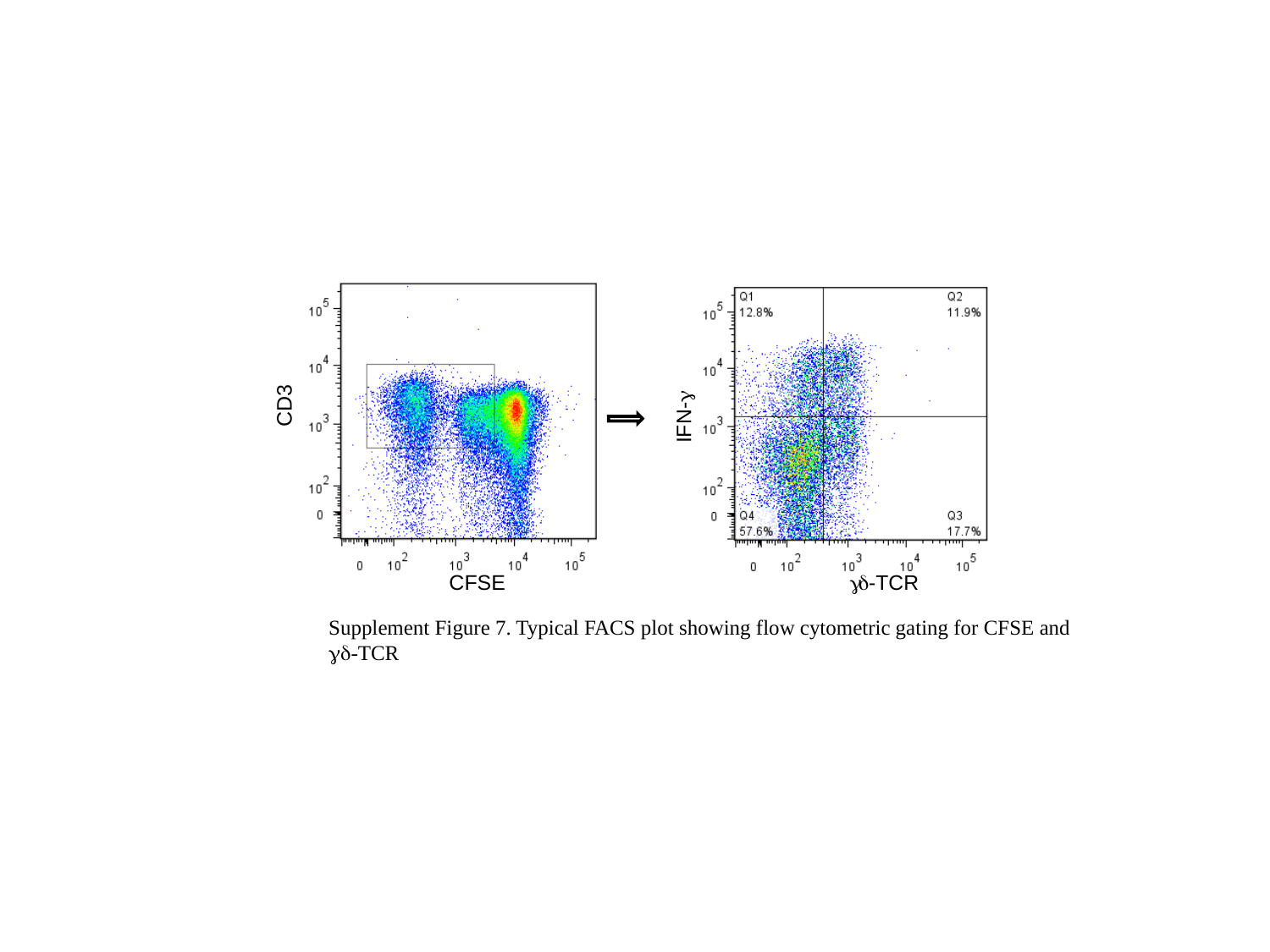

Supplement Figure 7. Typical FACS plot showing flow cytometric gating for CFSE and -TCR
